# Concentration-dependent dual activity of a phage-encoded lysin modulates infection dynamics in mycobacteriophage D29

**DOI:** 10.1128/mbio.00732-26

**Published:** 2026-07-13

**Authors:** Shinto James, Gokul Nair, Priyamvada Nath, Vikas Jain

**Affiliations:** 1 Microbiology and Molecular Biology Laboratory, Department of Biological Sciences, Indian Institute of Science Education and Research (IISER) 189785 , Bhopal, Madhya Pradesh, India; 2 Laboratory of Antimicrobial Innovation, School of Interwoven Arts and Sciences, Krea University 567852, Sri City, Andhra Pradesh, India; Montana State University, Bozeman, Montana, USA

**Keywords:** mycobacteria, bacteriophage, endolysin, phage therapy, tuberculosis

## Abstract

Lytic enzymes encoded by bacteriophages are conventionally understood to serve a single function in the phage life cycle: to enable progeny release through host cell lysis. Here, we identify an unexpected dual functionality in LysB, a lytic cassette protein of mycobacteriophage D29, which exerts opposing effects on phage propagation depending on the stage of infection. In our model, during the early phase of phage expansion, LysB’s activity is limited to infected cells, hydrolyzing their outer membrane to facilitate efficient virion release. However, as the infection progresses, LysB accumulates in the extracellular environment, eventually reaching levels sufficient to act on uninfected bystander cells, rendering them resistant to infection. Consistent with this model, deletion of *lysB* impairs progeny release at low multiplicity of infection yet enhances phage propagation at later stages of plaque expansion. We further show that exogenous treatment of host cells with LysB inhibits phage infection in a concentration-dependent manner, likely by removing cell-surface determinants required for phage adsorption. Together, these findings demonstrate LysB’s ability to modulate infection dynamics at the population level, revealing a potential new mechanism for phage-encoded host resilience. Engineered phages that lack this self-limiting activity may offer a path toward more effective antibacterial agents for therapeutic applications.

**IMPORTANCE:**

Lytic bacteriophages are often viewed as purely destructive viruses that rapidly eliminate their bacterial hosts. Yet, as in other predator–prey relationships, how these phages avoid exhausting their host populations remains poorly understood. Here, we report an example in which a phage-encoded protein that drives lysis also acts to limit it. LysB of mycobacteriophage D29 facilitates efficient virion release during infection but accumulates extracellularly as infection progresses, eventually blocking phage adsorption on uninfected cells in a concentration-dependent manner. The result is an intrinsic negative feedback on phage infection that might act to prevent host extinction. Given that rebounding infections pose a major challenge in the management of bacterial diseases, understanding and counteracting the mechanisms that underpin host resilience is crucial for the therapeutic application of phages.

## INTRODUCTION

Bacteriophages are key regulators of bacterial populations across diverse ecosystems. A typical phage life cycle begins with virion adsorption to specific receptors on the host cell envelope and concludes with the lysis of the same envelope, releasing progeny virions. Lysis is mediated by a tightly regulated, multi-step enzymatic program driven by phage-encoded lytic proteins that target specific structural components of the bacterial envelope. The substrate specificity and catalytic mechanisms of the lytic proteins vary widely among bacteriophages, reflecting the diversity of their bacterial hosts and the distinct architectures of their cell envelopes (1).

Phages that infect mycobacteria are particularly noteworthy due to the exceptional complexity of the host cell envelope they must breach for virion release. In addition to the cytoplasmic membrane and a heavily cross-linked peptidoglycan cell wall, mycobacteria possess an additional layer composed of branched arabinose and galactan polymers known as arabinogalactan. The terminal arabinofuranose residues of a subset of these arabinogalactan chains are esterified to long-chain mycolic acids (2), creating a covalent bridge to an outer lipid-rich membrane. Other lipids and lipoglycans, including trehalose mycolates, phospholipids, and glycopeptidolipids, intercalate the mycolic acid chains to form the outer membrane termed the mycomembrane (3, 4). The mycomembrane forms a highly effective permeability barrier, protecting mycobacteria from desiccation, chemical damage, and several antibiotics (3).

To overcome this multilayered barrier, mycobacteriophages encode dedicated lytic cassette proteins that act on specific layers of the mycobacterial envelope (5). Holins assemble within the cytoplasmic membrane to form pores that permeabilize the bilayer (6). The multidomain endolysin lysin A (LysA) hydrolyzes the peptidoglycan layer (7). Lysin B (LysB), a mycolylarabinogalactan esterase, cleaves the ester bond connecting arabinogalactan to the mycomembrane (8), releasing free mycolic acids. Thus, the coordinated activity of these proteins breaks down the mycobacterial envelope, enabling the efficient release of phage progeny.

Mycobacteriophage D29 is one of the earliest mycobacteriophages to be isolated (9) and characterized (10). It is a lytic phage capable of infecting several slow and fast-growing mycobacterial species (9, 11), including clinical isolates of *M. tuberculosis* (9). In the non-pathogenic laboratory model *M. smegmatis* (Msm), D29 has a life cycle of approximately 90 min (12). We have previously characterized the molecular properties and physiological relevance of D29 holin and LysA in *M. smegmatis* (13–15).

Unlike LysA, LysB is non-essential for mycobacteriophage propagation, as evidenced by the successful isolation of viable Giles and Ms6 phages lacking this enzyme (16, 17). However, the impact of LysB deletion varied between the phages. In Giles, *lysB* deletion resulted in a 30-min delay in the onset of ATP release and the entrapment of almost half of the phages in host cells (17). In Ms6, however, no delay in lysis timing was observed, but a significant number of phages were still trapped inside host cells, leading to a more than twofold decrease in burst size (16).

In this work, we attempted to elucidate the role of LysB in D29 mycobacteriophage physiology and its importance in phage propagation. We here show that the deletion of the *lysB* gene in D29 does not affect the timing of lysis but causes a marked reduction in burst size. The ΔLysB phage exhibits an altered plaque morphology in the later stages of plaque growth, which further prompted us to investigate the impact of extracellular LysB on phage infection. Remarkably, our findings reveal a multifaceted role for LysB in D29 physiology, where it functions as a population-level infection modulator, ultimately acting to promote host resilience in high host density environments. By uncovering this intrinsic, enzyme-mediated negative feedback that limits infection, our work reshapes current understanding of lytic phage ecology and provides insights for the rational design of more effective phage-based therapeutics.

## RESULTS

### LysB deletion does not alter the kinetics of D29-mediated host cell lysis

**Fig 1:**
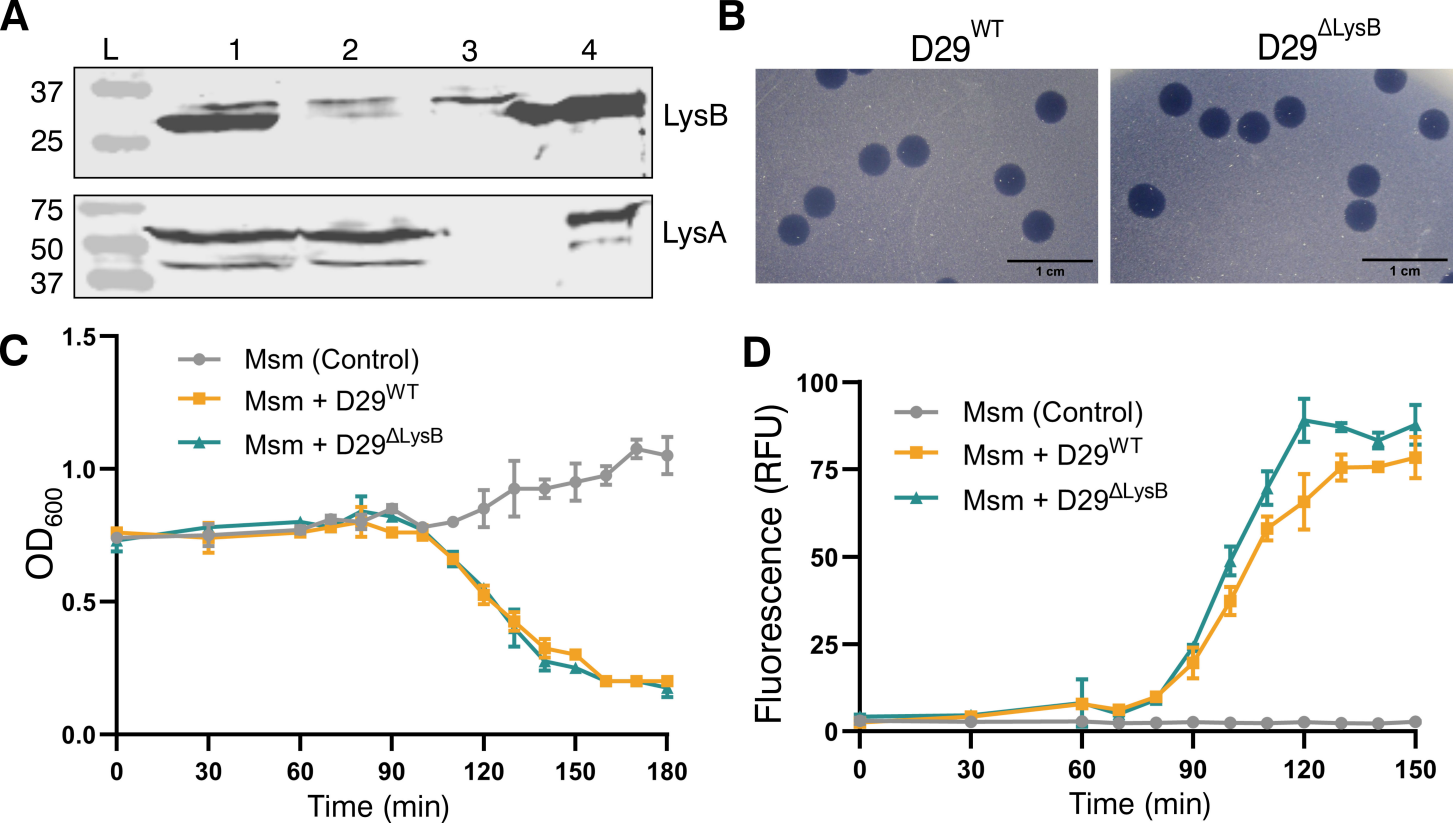
Validation of *lysB* deletion and its impact on D29 infection kinetics. (**A**) Western blots of Msm cells collected 60 min after phage infection (multiplicity of infection [MOI] = 3). Samples were probed with antibodies against LysB (upper panel) and LysA (lower panel). Lane 1 shows cells infected with D29^WT^, lane 2 shows cells infected with D29^ΔLysB^, lane 3 shows uninfected cells, and lane 4 shows purified LysA and LysB proteins from *E. coli* as controls. “L” indicates molecular weight standards with selected bands marked in kDa. (**B**) Plaques formed by D29^WT^ and D29^ΔLysB^ on a 16 h Msm lawn. Scale bar represents 1 cm. (**C**) Kill kinetics of log phase Msm cells after infection with either wild-type or mutant (D29^ΔLysB^) phage at MOI = 3. Optical density at 600 nm (OD_600_) was measured over time. Data are shown as mean ± SD from two independent experiments. (**D**) The plot shows GFP release following phage infection. Log-phase Msm cells expressing GFP from an integrative plasmid were infected with D29^WT^ or D29^ΔLysB^ at MOI = 3. GFP release was monitored by measuring fluorescence of culture supernatant at 525 nm. Data are shown as mean ± SD of three independent experiments.

To investigate the role of LysB in D29 physiology, we deleted the LysB-coding gene, *gp12*, from the D29 genome using the CRISPY-BRED method (18). In CRISPY-BRED, mutant phages are generated via recombineering and enriched by Cas9-mediated counterselection. In our attempt to delete *gp12* from the D29 genome, although we failed to isolate a full-length deletion mutant defined by the recombineering substrate, several deletion and frameshift mutants disrupting *gp12* were obtained in the counterselection background. We selected a mutant with a deletion spanning amino acids 52–247 (Fig. S1), which includes all three previously described active site residues of D29 LysB (17). Western blotting carried out 60 min post-phage infection confirmed that this mutant (D29^ΔLysB^) does not express LysB. In contrast, the expression of LysA was comparable to that of the wild-type D29 (D29^WT^) (Fig. 1A). We also observed no difference in plaque sizes between D29^ΔLysB^ or D29^WT^ after 16 h of plaque growth (Fig. 1B; Fig. S2).

To assess the effect of LysB deletion on the timing of D29-mediated host cell lysis, we monitored the growth of *M. smegmatis* infected with either D29^WT^ or D29^ΔLysB^ at an MOI of 3 (Fig. 1C). OD_600_ measurements show that both cultures follow similar growth profiles, suggesting that LysB deletion does not significantly affect the kinetics of host cell lysis. ATP release from the phage-infected cells, serving as an additional indicator of lysis, aligned with these results (Fig. S3), differing by fewer than 10 min in overall timing. To determine whether the release of a larger molecule from the cytoplasm might reveal subtle lysis defects, we expressed GFP in the host cells from an integrative vector and tracked its release over time following phage infection. GFP release profiles were also found to be similar between the two phages (Fig. 1D), confirming that LysB does not impact the timing of D29-mediated host cell lysis.

### LysB is essential for complete virion release in D29 infection

**Fig 2:**
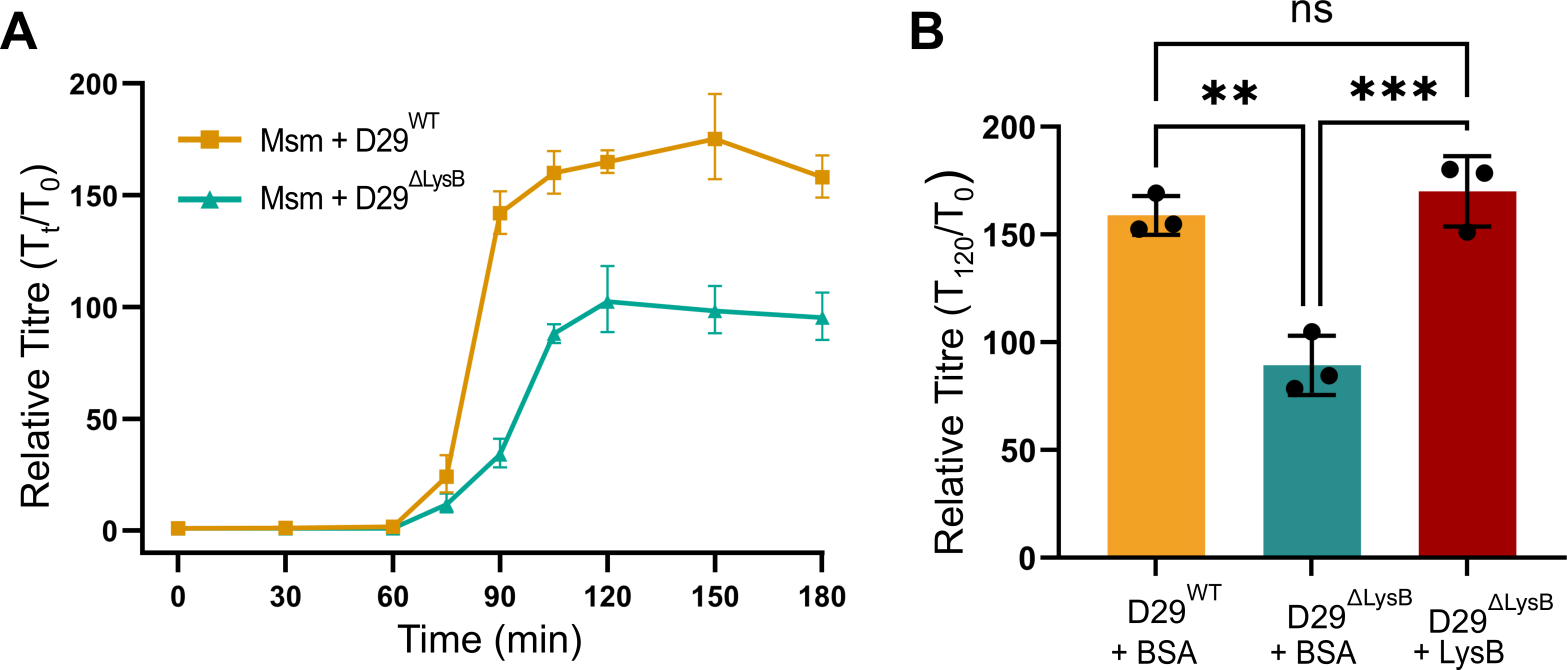
Contribution of LysB to D29 burst size in *M. smegmatis***.** (**A**) The plot shows the one-step growth curve of D29^WT^ and D29^ΔLysB^. T_*t*_/T_0_ is the relative phage titer, where T_*t*_ is the PFU/mL estimated at time *t*, and T_0_ is the PFU/mL estimated immediately after adsorption and removal of unadsorbed phages. Data are shown as mean ± SD from two independent experiments. (**B**) Estimation of the average burst size of D29^WT^ and D29^ΔLysB^ phages following treatment of infected cells with purified LysB or BSA as indicated. Burst size is calculated as PFU/mL at 120 min divided by PFU/mL at 0 min after adsorption. Data are shown as mean ± SD of three independent experiments. Statistical comparisons were performed using one-way ANOVA followed by Tukey’s multiple comparisons test. “ns,” not significant; “**,” *P* < 0.01; “***,” *P* < 0.001.

Next, we examined whether other aspects of phage growth, such as the burst size, were affected. A one-step growth curve revealed a markedly altered profile for D29^ΔLysB^, with a significant reduction in relative phage titer following burst, while the latent period and rise time remained unchanged (Fig. 2A). Whereas wild-type D29 exhibited an average burst size exceeding 150, the deletion of *lysB* reduced this to less than 100.

The reduced burst size indicated that a significant fraction of phage progeny remained trapped within incompletely lysed host cells. Since previous studies have shown that LysB can access its substrate on Msm when added exogenously (19), we reasoned that the lysis defect could be rescued by treating phage-infected cells with purified LysB. Based on data from the one-step growth curve, LysB was added at the onset of lysis (60 min), and titers were measured once the burst was complete (120 min). Remarkably, treating D29^ΔLysB^-infected Msm cells with exogenously-added 100 μg/mL LysB restored the burst size to wild-type levels (Fig. 2B). Together, these results demonstrate that in D29, similar to Ms6, the absence of LysB leads to incomplete host cell lysis without affecting the kinetics of lysis.

### LysB alters plaque morphology during the later stages of plaque expansion

**Fig 3:**
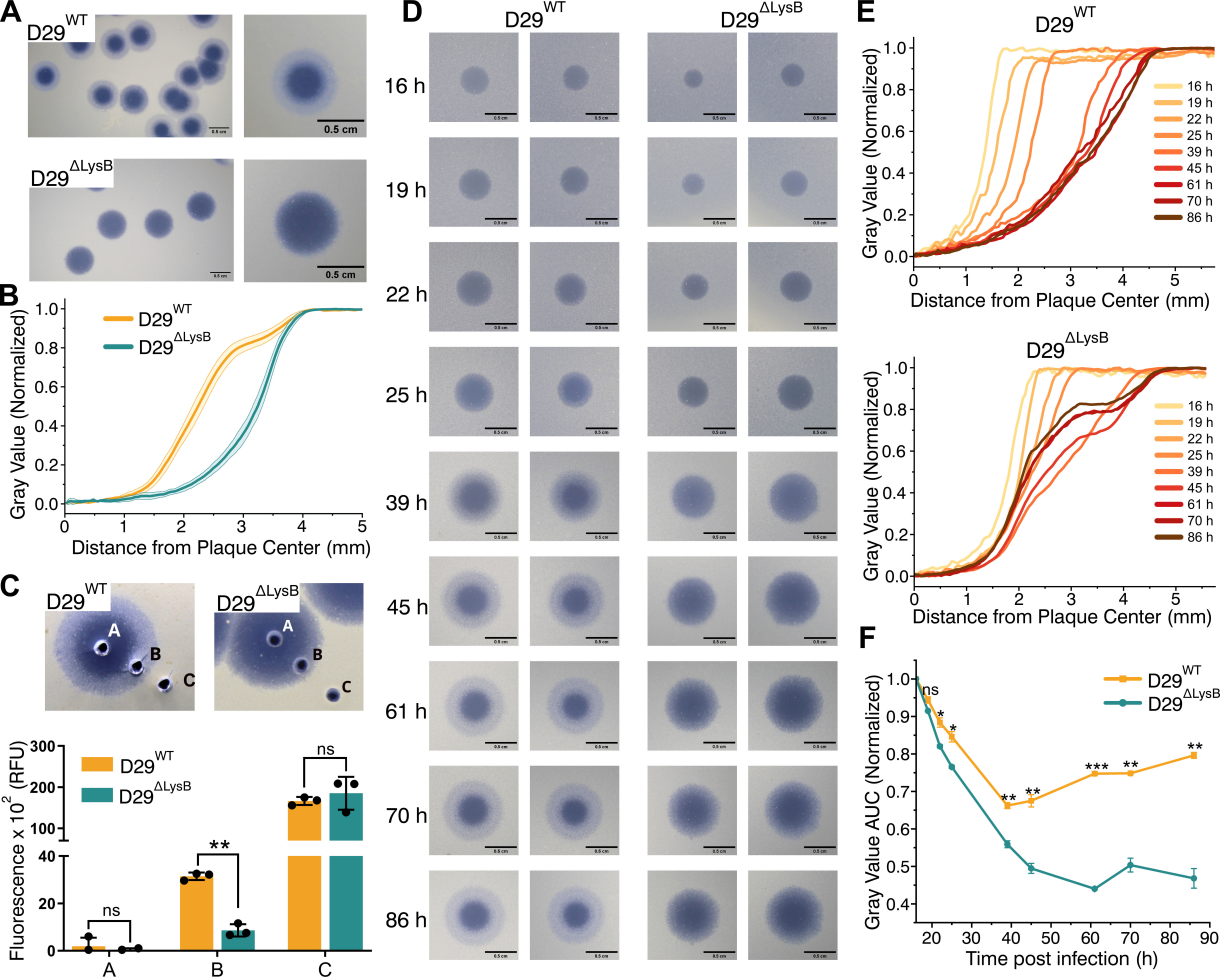
Role of phage-encoded LysB in late-stage plaque expansion. (**A**) Plaques formed by D29^WT^ or D29^ΔLysB^ on a 60 h lawn of Msm cells; scale bar, 0.5 cm. (**B**) Gray value intensity profiles measured radially from the plaque center outward for D29^WT^ and D29^ΔLysB^ plaques normalized to a 0–1 scale. Shaded areas represent the SD of three biological replicates. (**C**) Quantification of cell density in distinct regions of plaques formed by wild-type or D29^ΔLysB^ phage. Region A corresponds to the plaque center, region B to the plaque periphery, and region C to the surrounding lawn. Bar graphs show cell viability in each region as measured by the alamarBlue assay. Statistical significance was calculated by an unpaired t-test followed by Šídák-Bonferroni correction for multiple comparisons. “ns,” not significant; “**,” *P* < 0.01. (**D**) Tracking of plaque growth over time. The enlargement of plaques formed by D29^WT^ or D29^ΔLysB^ was monitored by photographing them at the indicated time points. (**E**) Gray value intensity profiles measured radially from the plaque center outward for D29^WT^ (top panel) and D29^ΔLysB^ (bottom panel) plaques at successive time points. Gray values were normalized to a 0–1 scale where 0 represents maximum clearing and 1 represents intact lawn density. Progressive rightward shifts in the curve indicate expansion of the lysis zone over time. (**F**) Quantification of lawn density over the infection time course using the area under the gray value profile (AUC) as a proxy metric. AUC values were calculated from the radial gray value profiles in panel E at each time point and normalized to the value at 16 h post-infection. A lower AUC corresponds to greater clearing of the bacterial lawn. Data represent the mean ± SD of three independent biological replicates. Statistical significance was determined by two-way ANOVA followed by Tukey’s multiple comparisons test. “ns,” not significant; “*,” *P* < 0.05; “**,” *P* < 0.01; “***,” *P* < 0.001.

The mycobacterial cell surface is reported to undergo modifications such as an increase in peptidoglycan cross-linking (20) and alterations in the cell wall and mycomembrane contents as the cells approach the stationary phase (4, 21). We, therefore, asked whether *lysB* deletion in D29 phage would show a more pronounced role in host lysis during the later stages of host growth. Bacteriophage plaques provide a convenient model for this, as the expanding plaque encounters a bacterial lawn progressing through the typical stages of the bacterial growth curve (22). Once nutrients in the growth medium are depleted, host cells enter the stationary phase, at which point most phages can no longer propagate, and plaque expansion ceases. To assess how LysB impacts phage propagation under these conditions, plaques were incubated well beyond the standard 16 h. Interestingly, when observed at 60 h post-incubation, D29^WT^ plaques displayed a distinct halo-like structure, whereas D29^ΔLysB^ plaques remained notably clearer with minimal peripheral turbidity (Fig. 3A). Densitometric analysis across plaque cross-section showed significantly greater turbidity in WT plaques compared to the LysB mutant (Fig. 3B). To estimate the viability of cells in this halo zone, we carried out an alamarBlue assay in this region by stab sampling (23–25). Our data indicate that the peripheral halo zone of D29^WT^ plaques harbors significantly more cells than that of D29^ΔLysB^ (Fig. 3C).

If LysB contributed to the lysis of late-stage Msm cells, the LysB-deficient phage would be expected to stop expanding once the plaques formed by the D29^WT^ began forming halos. However, very interestingly, our time-course tracking revealed the opposite outcome. After 24 h, D29^WT^ plaques slowed in growth and developed a turbid periphery, whereas D29^ΔLysB^ plaques continued expanding for more than 48 h, ultimately producing a substantially larger lysis zone than the WT phage (Fig. 3D). To quantify this, we performed densitometric analysis of plaque images (Fig. 3E) and used the area under the gray value profile (AUC) as a proxy for lawn density. D29^ΔLysB^ plaques showed a markedly greater reduction in lawn density compared to D29^WT^ as early as 22 h post-infection, with AUC values remaining significantly lower throughout the time course (Fig. 3F). Moreover, in contrast to the sustained lawn clearance seen with D29^ΔLysB^, D29^WT^ plaques recovered partial opacity after 40 h post-infection and continued growing thereafter, suggesting that LysB accumulation permitted bacterial rebound at later stages of infection.

**Fig 4:**
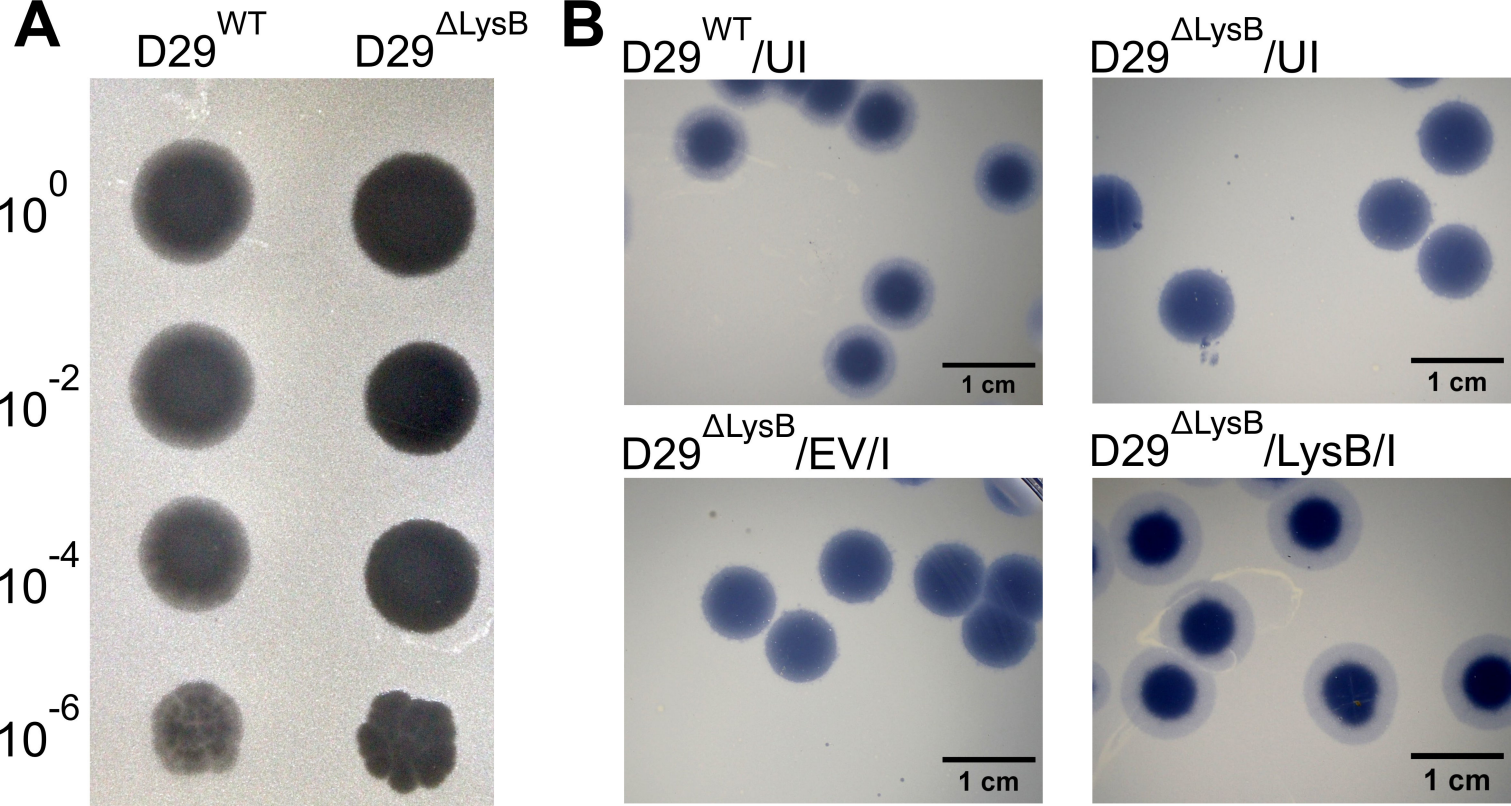
Loss and complementation of the halo phenotype in the D29^ΔLysB^ mutant. (**A**) Spotting of serial dilutions of D29^WT^ or D29^ΔLysB^ on a 14-h Msm lawn. Dilution factors are indicated. The lawn was imaged 60 h after phage spotting. (**B**) Complementation of the halo phenotype in D29^ΔLysB^ by the expression of LysB in host cells. The *lysB* gene was cloned into the integrative plasmid pMIT2_His under the control of a tetracycline-inducible promoter. “UI” denotes uninduced, and “I” denotes induced with 100 ng/mL of anhydrotetracycline (ATc). Panels show D29^WT^ propagated in uninduced cells (D29^WT^/UI), D29^ΔLysB^ in uninduced cells (D29 ^ΔLysB^/UI), D29^ΔLysB^ in empty vector cells induced with ATc (D29 ^ΔLysB^/EV/I), and D29^ΔLysB^ in LysB-expressing cells induced with ATc (D29^ΔLysB^ /LysB/I).

These observations suggested that the presence of LysB may negatively impact phage propagation, at least during the later stages of plaque development. Spotting phages at various concentrations on a 14-h bacterial lawn further confirmed this, showing that spots from D29^WT^ were markedly more turbid than those from the LysB-knockout phage (Fig. 4A). To validate the role of LysB in the halo formation, we complemented the deficit in D29^ΔLysB^ by expressing LysB from tetracycline (Fig. 4B) and acetamide (Fig. S4) inducible promoters in host cells. As expected, complementation restored the halo phenotype in the plaques. Furthermore, when propagated through these cells, a significant exaggeration of the peripheral turbidity was noticed, likely due to the overexpression of LysB from the strong promoters.

### Extracellular LysB limits phage propagation in a concentration-dependent manner

**Fig 5:**
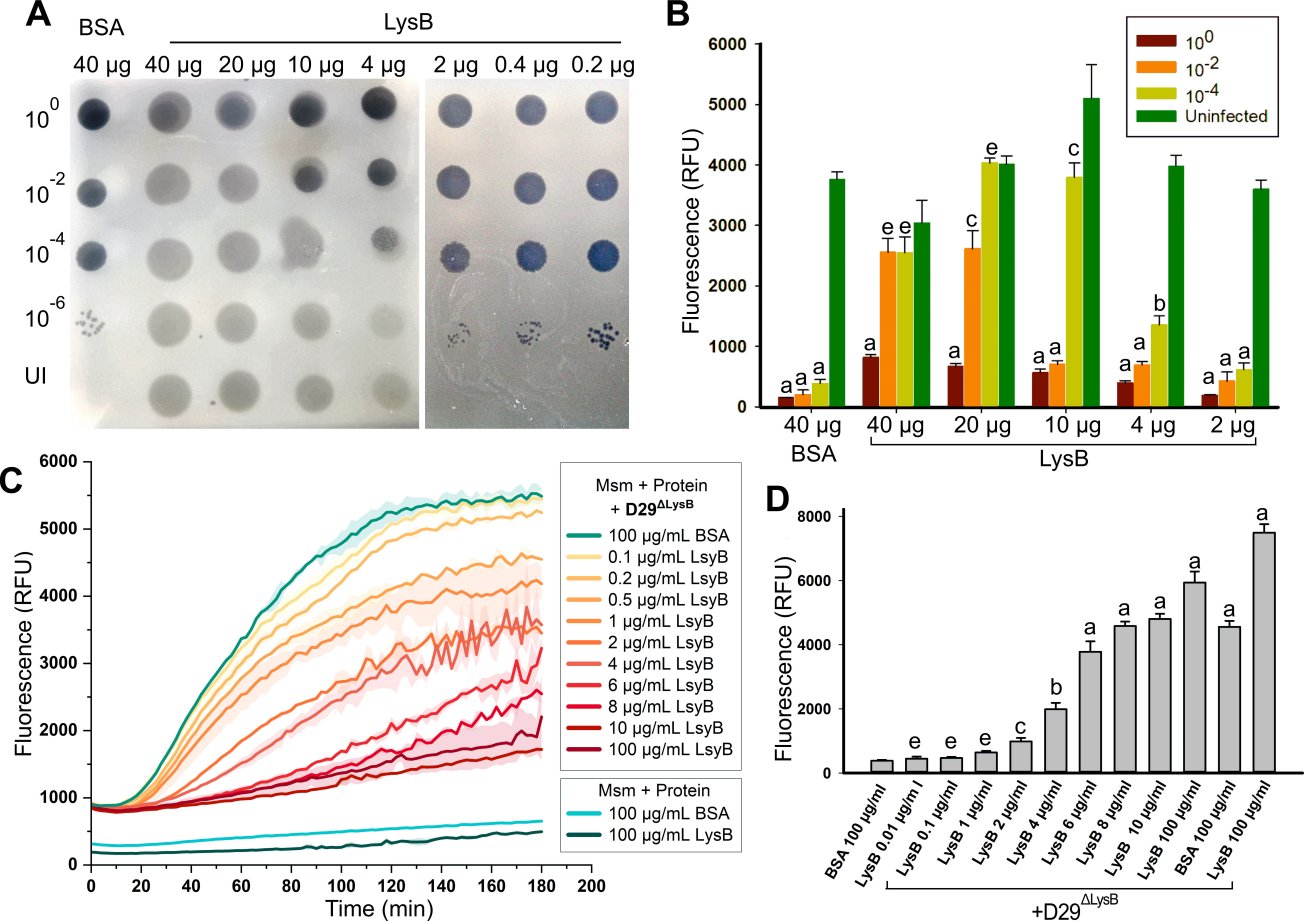
Inhibition of phage infection by extracellular LysB. (**A**) EOP of D29^ΔLysB^ on Msm cells treated with increasing concentrations of LysB. Log-phase Msm lawns were pretreated for 30 min with the indicated amounts of BSA or LysB by spotting 20 μL of each protein solution. Subsequently, 5 μL of D29^ΔLysB^ phage was spotted onto the protein-treated cells. Phage titers are indicated on the left. The 10^−6^ dilution corresponds to ~30 PFU. “UI” denotes uninfected. (**B**) Estimation of cell density at the protein-phage-treated spots from panel A. Stab samples (*N* = 3) were collected from each spot, resuspended in PBS, and cell density was quantified using the alamarBlue assay. The bar graph shows mean fluorescence from the reduced alamarBlue reagent. Statistical significance was determined by two-way ANOVA followed by comparing each condition to its corresponding uninfected control using Bonferroni’s multiple comparisons test “a,” *P* < 0.0001; “b,” *P* < 0.001; “c,” *P* < 0.01; “d,” *P* < 0.05; “e,” not significant. (**C**) SYTOX permeability assay to quantify phage-induced cell lysis. Planktonic Msm cells (OD_600_ = 0.8) were treated with the indicated concentrations of purified LysB for 30 min at 37°C, followed by infection with D29^ΔLysB^ at MOI = 3. The phage-infected samples were mixed with SYTOX dye, and fluorescence was monitored over time in a 96-well plate reader. The graph shows the average fluorescence of three biological replicates, with error bars representing standard deviation. Cells treated with 100 μg/mL BSA served as the negative control. (**D**) Assessment of cell viability in planktonic Msm cells following phage infection. Cells (OD_600_ = 0.8) were treated with varying concentrations of purified LysB for 30 min at 37°C in a multi-well plate. This was followed by infection with D29^ΔLysB^ at MOI = 3 and incubation at 37°C for 3 h. Cell viability was assessed using the alamarBlue assay. Equal volumes of alamarBlue reagent were then added to all samples, followed by incubation for 45 min at 37°C. The extent of alamarBlue reduction was quantified by measuring fluorescence (excitation 530 nm, emission 590 nm). Statistical comparisons were performed using one-way ANOVA followed by Tukey’s multiple comparisons test. “a,” *P* < 0.0001; “b,” *P* < 0.001; “c,” *P* < 0.01; “d,” *P* < 0.05; “e,” not significant.

LysB-mediated turbidity confined to the periphery of the plaques suggested two possibilities. First, LysB can only inhibit phage infection during the late phase of Msm growth. Alternatively, LysB could act independently of the host growth phase but requires accumulation to a threshold extracellular concentration to induce phage “resistance,” a level reached only toward the end of plaque expansion. To test this, we treated an actively growing Msm lawn with varying concentrations of LysB for 30 min and then spotted serial dilutions of D29^ΔLysB^ phage onto the treated cells to measure the efficiency of plating (EOP). D29^ΔLysB^ was used here to prevent interference from LysB carried over in the wild-type phage lysate. For cells treated with up to 4 μg of LysB, we observed a partial reduction in lawn turbidity, even in the absence of any phage (Fig. 5A). However, quantification of stab samples taken from these uninfected (UI) zones showed that the partial loss of turbidity did not correspond to a reduction in cell density (Fig. 5B). This is consistent with previous studies demonstrating that LysB treatment of Msm in the absence of surfactants does not impair cell growth (8, 19, 26, 27). We instead propose that the reduced turbidity reflects LysB-mediated removal of outer-membrane lipids, which constitute a substantial fraction of the mycobacterial cell-wall mass (28). This resembles the action of phage depolymerases, which remove bacterial capsules or exopolysaccharides, thereby reducing light scattering and decreasing the turbidity of the lawn without compromising cell viability (29–31).

Very interestingly, spotting phage onto the LysB-treated regions inhibited phage propagation in a concentration-dependent manner, as shown by the phages’ inability to further reduce turbidity (Fig. 5A). This was further validated by alamarBlue quantification of the LysB-phage co-treatment zones (Fig. 5B). Our data show that increasing LysB concentrations enabled Msm cells to resist progressively higher phage titers, demonstrating a concentration-dependent protective effect of LysB.

We next asked whether LysB induces a similar phenotype in planktonic Msm. To examine this, cells were treated with varying concentrations of LysB for 30 min at 37°C and then infected with D29^ΔLysB^ at an MOI of 3. Phage-mediated lysis was monitored by measuring OD_600_ in a multiwell plate (Fig. S5). While the BSA-treated control showed a sharp decline in OD, LysB-treated samples displayed partial protection starting at 2 μg/mL of LysB, and remained completely unlysed above 8 μg/mL. This resistance was further confirmed using a SYTOX Green uptake assay, which reports phage-induced membrane compromise (32). SYTOX measurements reveal a similar concentration-dependent inhibition of lysis following LysB treatment (Fig. 5C). To determine whether the unlysed cells remain viable, we measured cell viability using an alamarBlue assay 3 h post-infection. LysB treatment resulted in a concentration-dependent protection from phage-induced killing, evidenced by a progressive increase in the pink-colored resorufin production with higher LysB concentrations (Fig. 5D).

Interestingly, we also noticed that the treatment with LysB alone increased alamarBlue fluorescence (Fig. 5D). To examine this further, we exposed Msm cells to varying concentrations of LysB for 3 h and then performed a real-time alamarBlue assay. LysB treatment led to a concentration-dependent increase in alamarBlue reduction (Fig. S6). Because the mycomembrane is a major barrier to nutrient influx in mycobacteria (33, 34), this likely reflects increased metabolism or cell growth resulting from improved nutrient access following LysB-mediated compromise of the mycomembrane. Further studies are ongoing to investigate this phenomenon.

### Extracellular LysB inhibits phage infection at the adsorption step

**Fig 6:**
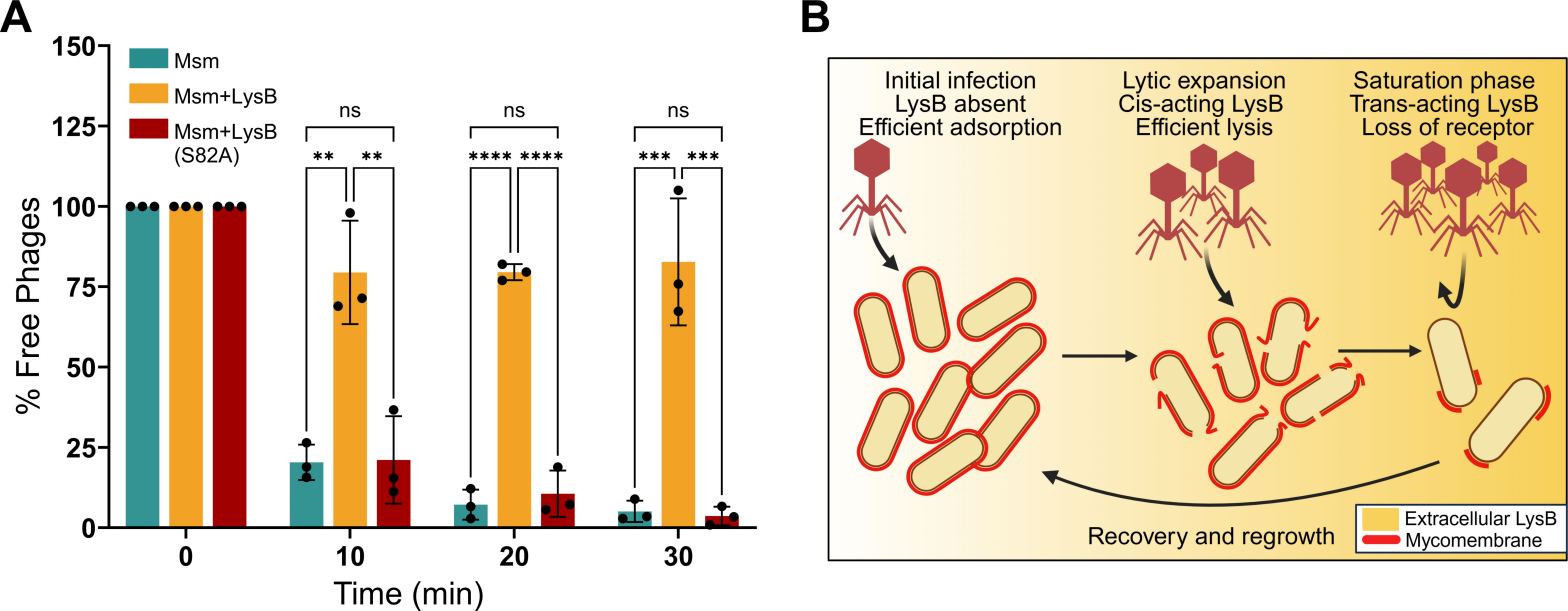
Impact of LysB on D29 adsorption and its broader role in phage ecology. (**A**) Quantification of D29 phage adsorption on LysB-treated Msm cells. Log-phase cells were treated with 50 μg/mL LysB for 30 min, followed by the addition of D29 phage. Adsorption at the indicated time points was measured by quantifying the number of unabsorbed phages remaining in the supernatant. Data are presented as mean ± SD of three independent experiments. Statistical significance was calculated by one-way ANOVA followed by Bonferroni’s multiple comparisons test at each time point. “ns,” not significant; “**,” *P* < 0.01; “***,” *P* < 0.001; “****,” *P* < 0.0001. (**B**) Schematic of the proposed model where LysB exerts opposing effects on phage propagation in a concentration-dependent manner. In the absence of extracellular LysB, phages adsorb efficiently to bacterial surface receptors, initiating infection. During the early stages of lytic expansion within the population, LysB’s activity is limited to the producer cells, facilitating efficient host cell lysis. As infection approaches saturation, extracellular LysB builds up to higher concentrations, removing surface receptors on bystander cells. This limits further phage adsorption, leaving behind a small fraction of surviving cells. In natural environments, phages may diffuse away from the immediate site of lysis, allowing surviving cells to recover and reestablish the population. Thus, LysB functions as a concentration-dependent regulator, creating a self-limiting infection that balances phage propagation with host population resilience.

Given that LysB targets the outermost membrane of mycobacteria, we hypothesized that it mediates phage resistance by selectively removing bacterial surface receptors. To test this, we performed a phage adsorption assay on Msm cells treated with purified LysB. An inactive LysB mutant was used as a control, in which the serine residue (S82) of the catalytic triad (S82, D166, and H240) was substituted with alanine (17, 35). Loss of esterase activity was confirmed by a p-nitrophenyl butyrate (p-NPB) hydrolysis assay (Fig. S7). Data from the adsorption assay show that treatment of Msm cells with 50 μg/mL LysB for 30 min markedly inhibits phage adsorption, with over 80% of phages remaining unbound. In contrast, untreated cells and cells treated with a catalytically inactive mutant of LysB exhibited rapid adsorption, with more than 80% of phages bound within 10 min (Fig. 6A).

Together, these findings demonstrate the double-edged nature of LysB present in D29 phage on its propagation, enabling it to act as a population-level phage infection modulator. We summarize this in a proposed model of LysB-mediated phage dynamics (Fig. 6B). At early stages of infection, LysB promotes phage propagation by efficiently hydrolyzing the mycobacterial cell wall, leading to increased burst size. However, as infection advances and MOI rises, the enzyme accumulates extracellularly, where it strips the host of surface phage receptors and slows adsorption, thereby reducing the likelihood of superinfection and facilitating broader virion dispersal. With continued accumulation, LysB ultimately abolishes phage adsorption altogether, sparing the bacterial population from infection. By removing the mycomembrane that functions as a permeability barrier to nutrients, LysB also facilitates rapid regrowth of these survivors and re-establishment of the population. The result is a form of viral self-regulation that stabilizes host-virus dynamics and preserves long-term host availability.

## DISCUSSION

As antibiotic-resistant infections emerge as a global health emergency, bacteriophages and phage-derived enzymes present a promising therapeutic alternative. Lytic phages can rapidly collapse bacterial populations, and early trials and laboratory studies consistently highlight their ability to initiate clearance (36). Yet, recurrence of infection is frequently observed, as small numbers of surviving cells reseed the population and the infection rebounds (37–39). Interestingly, from an ecological perspective, such recurrence benefits the phage, ensuring long-term coexistence with its host. Temperate phages achieve this balance through a highly regulated life cycle that switches between lysis and lysogeny, coordinated with host population density (40). By contrast, how strictly lytic phages avoid exhausting their host populations remains obscure.

In this study, we examined the role of a phage-encoded lytic factor, LysB, in modulating infection dynamics by mycobacteriophage D29 in *Mycobacterium smegmatis*. In most sequenced mycobacteriophages, *lysB* is found within the lytic cassette of the genome (7), and the gene product is shown to function as a mycolylarabinogalactan esterase, cleaving the ester linkage between mycolic acid and the arabinogalactan in the mycobacterial cell wall (17). Although LysB-deficient mycobacteriophages have been previously isolated and characterized (16, 17), the specific contribution of LysB to D29 infection had remained unclear.

The defective virion release observed in the LysB knockout D29 phage highlights the crucial role of LysB in D29-mediated cell lysis. Interestingly, unlike the LysB mutant in Giles, which shows a clear delay in lysis (17), D29^ΔLysB^ did not display any detectable shift in lysis timing, resembling instead the behavior reported for the Ms6 LysB mutant (16). The fraction of trapped virions in D29^ΔLysB^ is also lower than what has been reported for both Giles and Ms6 (16, 17). This likely explains why the D29 mutant did not show a decrease in plaque size, unlike the reductions in plaque size observed for the other two phages. Overall, these results indicate that LysB contributes to efficient host cell lysis, but its relative significance differs among mycobacteriophages.

The observation that the LysB-deficient phage propagated more efficiently through a maturing lawn was contrary to our expectations. The increased turbidity at the edges of D29^WT^ plaques suggested two possibilities. Either the WT phage was intrinsically unable to infect these late-stage cells, or LysB released during earlier rounds of infection rendered the bacterial lawn resistant to further infection. The restoration of the halo-like phenotype when D29^ΔLysB^ was propagated through LysB-expressing cells, along with the significant impairment of phage propagation by treatment of the bacterial lawn with purified LysB, supported the latter explanation. Furthermore, the ability of LysB to inhibit phage infection in a concentration-dependent manner in planktonic cultures provided definitive evidence for this hypothesis.

The reduced phage adsorption observed following LysB treatment suggests that the resulting phage resistance arises from the removal or alteration of surface determinants required for D29 binding. Although the exact receptor for D29 has not been defined, its broad host range suggests that it targets an abundant, conserved surface moiety (41); the ability of LysB to remove this component from the cell envelope may offer a potential strategy for identifying the D29 receptor on mycobacterial cells.

The varying degrees of impairment observed across D29, Giles, and Ms6 LysB mutants are perhaps unsurprising given the high sequence and structural differences among their respective LysB proteins. LysB proteins, in general, show high diversity across mycobacteriophages (7). In particular, significant differences in the loop-5 motif that harbors the catalytic histidine residue, producing a wide diversity of active site conformations, have been noted (26). Additionally, the extended N-terminal peptidoglycan-binding domain present in Ms6 LysB is absent in several other homologs, a difference that could influence substrate accessibility and the dynamics of enzyme release following lysis (7, 17). Given this diversity, we are cautious about generalizing the concentration-dependent inhibitory activity we describe here to other LysB family members and restrict our conclusions to D29. Whether this property is unique to D29 LysB or is more broadly shared across the family is an interesting question that needs further experimental investigation.

Overall, our findings demonstrate the double-edged impact of LysB in mycobacteriophage D29 propagation. At the population level, LysB-mediated phage resistance might serve to maintain the equilibrium of phage-bacterial population, and, therefore, community stability by preventing the extinction of host bacteria. At the same time, LysB’s positive impact on burst size explains how the gene is maintained in the phage population, preventing the emergence of LysB-deficient defectors. However, whether this dual functionality reflects an evolved regulatory mechanism for phage-host coexistence or simply an off-target effect of LysB as it accumulates to high concentrations beyond its primary site of action remains an open question. It is also to be noted that the inhibitory role of LysB was strictly concentration-dependent and was observed in high-density host lawns, where LysB concentrations can reach high levels during infection. Future studies examining LysB behavior across a range of host densities will be critical for determining whether the population-level feedback we describe is a general feature of D29 infection or if it is specific to high-density conditions.

From a practical standpoint, our engineered phage lacking LysB eliminates this self-limiting safeguard, thereby sustaining a more aggressive infection that minimizes bacterial survival *in vitro*. Whether sufficient extracellular LysB accumulates *in vivo*, where bacterial densities are considerably lower, and protein pharmacokinetics differ substantially to exert a similar inhibitory effect, remains unclear. Nonetheless, our findings provide a rational basis for engineering phages with potentially enhanced lytic efficiency and highlight the importance of fully characterizing the functional repertoire of phage lytic proteins beyond their canonical role in host cell lysis.

## MATERIALS AND METHODS

### Bacterial strains, media, and growth conditions

*M. smegmatis* strain mc^2^155 was cultured in Middlebrook 7H9 (MB7H9) (Difco) medium supplemented with 2% glucose and 0.05% Tween 80 at 37°C with constant shaking at 200 rpm. For experiments involving phage infection, host cells were grown in MB7H9 supplemented with 2% glucose, 4% OADC (oleic acid, albumin, dextrose, and catalase; Difco), and 1 mM CaCl_2_. Solid culture medium was prepared by adding 1.5% agar and 2% glucose to the MB7H9 growth medium. For soft agar overlay experiments, 0.75% agar mixed in a growth medium was used. All cloning experiments were carried out in the E. *coli strain* XL1-Blue (Stratagene), whereas *E. coli* BL21(DE3) (Lucigen) was used for protein expression and purification. *E. coli* strains were grown in LB broth (Difco) supplemented with 100 μg/mL ampicillin and at 37°C with constant shaking at 200 rpm. Phage stocks were prepared by generating plaques on an MB7H9 agar plate using a soft agar overlay and collecting the phages from the plaques by letting them diffuse to the phage dilution buffer (10 mM Tris-HCl, pH 7.5, 10 mM MgSO_4_, 68.5 mM NaCl, and 1 mM CaCl_2_). Phages were stored in phage dilution buffer at 4°C

### PCR, molecular cloning, and site-directed mutagenesis

PCRs were carried out using Phusion high-fidelity DNA polymerase (NEB) using primers listed in Table S1. *lysB* was inserted at the EcoRV site of pMIA_His and pMIT2_His using a one-step cloning strategy described elsewhere (42). Plasmid constructs generated or used in this study are mentioned in Table S2. For generating the inactive mutant of the LysB protein, namely S82A, the transfer PCR method was employed, as described elsewhere (43) using the primers mentioned in Table S1.

### Expression and purification of proteins

Plasmids pET-21b_lysB and pET-21b_lysB(S82A) were used to express LysB and catalytically inactive LysB(S82A) mutant, respectively, as described previously (44, 45). To purify these proteins, *E. coli* BL21(DE3) harboring respective plasmids was grown at 37°C with constant shaking at 200 r/min and induced with 0.3 mM IPTG when the culture reached OD_600_ ∼0.6. The induced culture was further incubated at 22°C with constant shaking at 150 r/min for 8 h. Cells were then harvested and resuspended in lysis buffer (40 mM Tris-Cl, pH 8.0, 200 mM NaCl, 5 mM imidazole, 5 mM 2-mercaptoethanol). The resuspended cells were further lysed by sonication. The lysate was then centrifuged at 18,000 rpm at 4°C for 1 h, and the collected supernatant was incubated with pre-equilibrated Ni-NTA (Qiagen) beads for 2 h at 4°C. The matrix was then subjected to washing with a buffer containing 40 mM Tris-Cl, pH 8.0, 500 mM NaCl, 25 mM imidazole, and 5 mM 2-mercaptoethanol, and finally, the protein was eluted using a buffer containing 40 mM Tris-Cl, pH 8.0, 200 mM NaCl, 300 mM imidazole, and 5 mM 2-mercaptoethanol. The collected elutions were subjected to dialysis against a buffer having 40 mM Tris-Cl, pH 8.0, 200 mM NaCl, 1 mM dithiothreitol, and 40% glycerol. The dialyzed protein was centrifuged at 14,000 rpm for 30 min at 4°C, and stored at −20°C until further use.

### Substrate-dependent enzymatic activity of LysB

The enzyme activity of the LysB and its inactive mutant was monitored using p-nitrophenyl butyrate (p-NPB) as a substrate with slight modification (17, 46). Briefly, in a 200 μL reaction volume, 100 μg of protein was added to 10 mM of p-NPB (dissolved in DMSO) and incubated at 37°C for 15 min. Bovine serum albumin and LysB were used as negative and positive controls, respectively, while the sample without protein was kept as a blank. The release of p-nitrophenyl by hydrolysis of the substrate was monitored by recording the absorbance at 420 nm.

### Gene knockout in phage using CRISPY-BRED

D29^ΔLysB^ was generated using the CRISPY-BRED technique. CRISPY-BRED was carried out as described previously (18) but with slight modifications. A recombineering strain was generated by transforming *M. smegmatis* mc^2^155 with the plasmid pJV53_Hygro. pJV53_Hygro was developed from the pJV53 plasmid (Addgene plasmid #26904) by replacing the kanamycin resistance gene with the hygromycin resistance gene. Induced electrocompetent cells were prepared from the recombineering strain. Briefly, a 0.8 OD_600_ secondary culture of the recombineering strain was induced with 1% acetamide and incubated at 37°C, 200 rpm for 3 h. Following this, the cells were washed three times in ice-cold 10% glycerol and resuspended in 10% glycerol at 5% original volume, and flash-frozen in liquid nitrogen.

Approximately 500 bp of the upstream and downstream region of *lysB* was amplified from the D29 genome and joined together by overlap extension PCR to generate the recombineering substrate. The first 6 bp and last 62 bp of *lysB* were left intact in the recombineering substrate to avoid any polar effects from *lysB* deletion. For counterselection, a guide RNA corresponding to a deleted region in *lysB* was designed such that the CRISPR/Cas interference machinery only targets the wild-type D29. This gRNA cassette was inserted into pCRISPRx-Sth1Cas9-L5 (Addgene plasmid #140993) as described here (47). pCRISPRx-Sth1Cas9-L5 expresses the *S. thermophilus* Cas9 protein from a tetracycline-inducible promoter. *M. smegmatis* mc^2^155 was transformed with the above construct to generate a counterselection strain.

The recombineering cells were electroporated with 200 ng of D29 gDNA and 400 ng of recombineering substrate. Recovery media containing MB7H9 supplemented with 2% glucose, 4% OADC, and 1 mM CaCl_2_ was added, and the cells were incubated at 37°C, 200 rpm for 4 h to permit phage release. A secondary culture of the selection strain was induced with anhydrotetracycline (ATc) at 100 ng/mL concentration at ~0.8 OD_600_ and was incubated at 37°C, 200 rpm for 1 h. The recovered recombineering cells containing released phages were mixed with an equal volume of pre-induced counterselection cells. Following a 20-min adsorption at 37°C, the mixture was plated over MB agar plates through a soft agar overlay. These plates were supplemented with 2% glucose, 50 μg/mL kanamycin, and 100 ng/mL ATc.

The following day, plaques obtained in the above plate were cored out using a wide-bore pipette and resuspended in phage dilution buffer. An aliquot from the resuspended phages was boiled for 10 min and used as a template for a PCR to screen for desired mutants. Plaques identified as carrying the desired *lysB* deletion were further propagated, followed by a second round of plaque purification to isolate single plaques and ensure genetic homogeneity. The precise deletion junctions were confirmed by Sanger sequencing of the PCR amplicon spanning the *lysB* locus.

### Western blot analysis

*M. smegmatis* culture at OD_600_ ~0.8 was infected with the wild type or knockout phage at MOI = 3. Phage adsorption was carried out for 10 min at 37°C, following which the infected cells were incubated at 37°C, 200 rpm. Sixty minutes post-infection, cells were resuspended in urea lysis buffer (40 mM Tris-Cl, pH 8.0, 200 mM NaCl, 5 mM imidazole, 5 mM 2-mercaptoethanol, and 8 M urea). Following resuspension, the cells were lysed by sonication. The lysate was centrifuged at 14000 rpm at 4°C for 30 min. The supernatant was mixed with SDS-loading dye and was resolved on a polyacrylamide gel through electrophoresis. Western blotting was carried out using anti-LysB or anti-LysA antibodies. Subsequently, the samples were probed with anti-rabbit IgG DyLight 800 conjugated secondary antibody (Invitrogen). The blots were scanned on an Odyssey infrared imaging system (LI-COR Biosciences, Lincoln, NE).

### Cell lysis and cell viability assays

Phage-mediated host cell lysis was monitored by estimating the OD_600_ of the planktonic culture. Optical density in multiwell plate readings was recorded in a SpectraMax M5 plate reader (Molecular Devices). The ATP released into the culture supernatant following cell lysis was quantified using the BacTiter-Glo Microbial Cell Viability Assay kit (Promega), as previously described (25). GFP release assay was carried out using *M. smegmatis* harboring pMIA_GFP; this is an integrative mycobacterial vector with a gene coding for GFP under the regulation of an acetamide-inducible promoter. The secondary culture of this strain was induced with 0.2% acetamide for GFP expression. After 3 h of induction, the cells were infected with D29^WT^ or D29^ΔLysB^ at 3 MOI. A total of 500 μL of samples was collected at each time point, harvested at 10,000 rpm, and stored at 4°C. GFP fluorescence was quantified by measuring the emission at 525 nm following excitation at 488 nm in a SpectraMax M5 plate reader (Molecular Devices). Cell viability subsequent to phage infection was quantified by a microtiter alamarBlue (BIO-RAD) assay as previously described (25). Phage-induced bacteriolytic activity was also monitored in real time by quantifying the permeability to the SYTOX-green stain (Thermo Fisher Scientific). SYTOX-green staining solution was mixed with phage-infected cells in a multiwell plate. The fluorescent signal of the dye was measured every 2 min in a SpectraMax M5 plate reader (Molecular Devices). The temperature of the plate reader was maintained at 37°C, and the plate was shaken for 5 s before every read.

### One-step growth curve

A one-step growth curve was performed as previously described (25) with slight modifications. In total, 1 mL of 0.8 OD_600_ *M. smegmatis* was infected with phage at 1 MOI. Phage adsorption was allowed by incubating the cells at 37°C for 10 min. Unadsorbed phages were inactivated by the addition of 100 μL of 0.4% H_2_SO_4_. After incubation with H_2_SO_4_ for 10 min at 37°C, equinormal amounts of NaOH were added for neutralization. Following this, the samples were diluted in growth media to a concentration of 1,000 phage-infected cells per mL. This sample was incubated at 37°C, 200 rpm. One hundred microliters of the sample was aliquoted at each time point and mixed with 500 μL of log phase *M. smegmatis*, kept for phage adsorption for 10 min, and plated using soft-agar overlay. Samples collected after 75 min were diluted 10 or 100 times in phage dilution buffer before mixing with *M. smegmatis*. Phage titer at each time point was calculated by counting the plaques on the following day.

### LysB complementation—burst size

*M. smegmatis* culture at 0.8 OD_600_ was infected with D29^WT^ or D29^ΔLysB^ at 1 MOI. Following a 10-min adsorption phage, infected cells were incubated at 37°C, 200 rpm. After 60-min incubation, phage-infected cells were either exposed to 100 μg/mL of LysB or BSA and incubated for 60 more minutes. Subsequently, the release phages were serially diluted, and plaque-forming units were quantified by the soft agar overlay method.

### LysB complementation—plaque morphology

*M. smegmatis* was transformed with pMIA-His_lysB or pMIT2-His_lysB. The culture of each strain at OD_600_ ~0.8 was infected with 30 PFUs of D29^WT^ or D29^ΔLysB^. The bacterial suspension was mixed with soft agar and poured on a solid agar plate supplemented with or without the respective inducers (100 ng/mL of anhydrotetracycline for pMIT2-His_lysB and 0.2% acetamide for pMIA-His_lysB). Plates were incubated at 37°C for 60 h before imaging.

### Densitometric analysis of plaques

Plaque images were captured under consistent lighting conditions and analyzed using the radial profile plugin in ImageJ. For each plaque, a circular region of interest was drawn centered on the plaque, and gray value intensity was measured radially outward from the plaque center to the surrounding intact lawn. The resulting gray value profiles were normalized to a 0–1 scale where 0 represents the minimum intensity within the cleared zone, and 1 represents the maximum intensity of the intact bacterial lawn. For time-course analysis, the area under the gray value profile (AUC) at each time point was calculated (from the center to the edge of the plaque right before saturation), normalized to the first time point, and was used as a proxy for lawn density.

### Cell density estimation in the bacterial lawn

To estimate cell density in the bacterial lawn, a cylindrical slice of the agar with the bacterial lawn was cored out from the soft-agar overlay plate using a capillary glass tube (Warner Instruments, inner diameter: 0.86 mm). The agar sample was aspirated into the capillary tube using a pipette and thereafter resuspended in 90 μL of PBS in a multiwell plate. For an experiment, a single capillary tube was used to minimize variations in the capillary radius. The capillary tube was cleaned between samples by aspirating from an empty agar plate. Ten microliters of alamarBlue reagent was added to the stub samples in PBS and incubated at 37°C for 3 h. Viable cells were quantified by measuring fluorescence at 488 nm in a SpectraMax M5 plate reader.

### Adsorption assay

To quantify the impact of LysB on phage adsorption, LysB-treated *M. smegmatis* cells were subjected to a phage adsorption assay as previously described (14, 48). Briefly, log phase *M. smegmatis* cells were treated with 50 μg/mL of LysB or LysB(S82A) for 30 min at 37°C. The cells were then infected with D29 phage at MOI = 1 in the presence of 1 mM CaCl_2_. Aliquots of 1 mL culture were drawn every 10 min and centrifuged at 14,000 rpm at 4°C for 5 min. To determine the free phage titer, 100 μL of the resulting supernatant was further subjected to infection with 200 μL of Msm culture and plating the resulting mixture onto MB7H9 agar plates using the soft agar overlay method. Post 12-h incubation at 37°C, the plaques thus obtained were enumerated. Finally, the percentage of free phages was plotted against time, with the 0 min time point indicating 100% free phages and no phage adsorption.

### Statistical analysis

Statistical analyses such as Student’s t-test and analysis of variance (ANOVA) were carried out using GraphPad Prism 7.0 software (GraphPad Software, Inc., San Diego, CA, USA).

## SUPPLEMENTAL MATERIAL

Supplemental Figures mbio.00732-26-s0001.pdf Figures S1 to S7.

Supplemental Tables mbio.00732-26-s0002.pdf Tables S1 and S2.

ASM does not own the copyrights to Supplemental Material that may be linked to, or accessed through, an article. The authors have granted ASM a non-exclusive, world-wide license to publish the Supplemental Material files. Please contact the corresponding author directly for reuse.
