# Supplementary material for: Concentration-dependent dual activity of a phage-encoded lysin modulates infection dynamics in mycobacteriophage D29
Source: mBio. 2026 Jul 13;17(8):e00732-26. doi: 10.1128/mbio.00732-26 (PMC13463830; doi:10.1128/mbio.00732-26)
Supplement: Supplementary file 1 [file mbio.00732-26-s0001.pdf]

Supplementary Figures

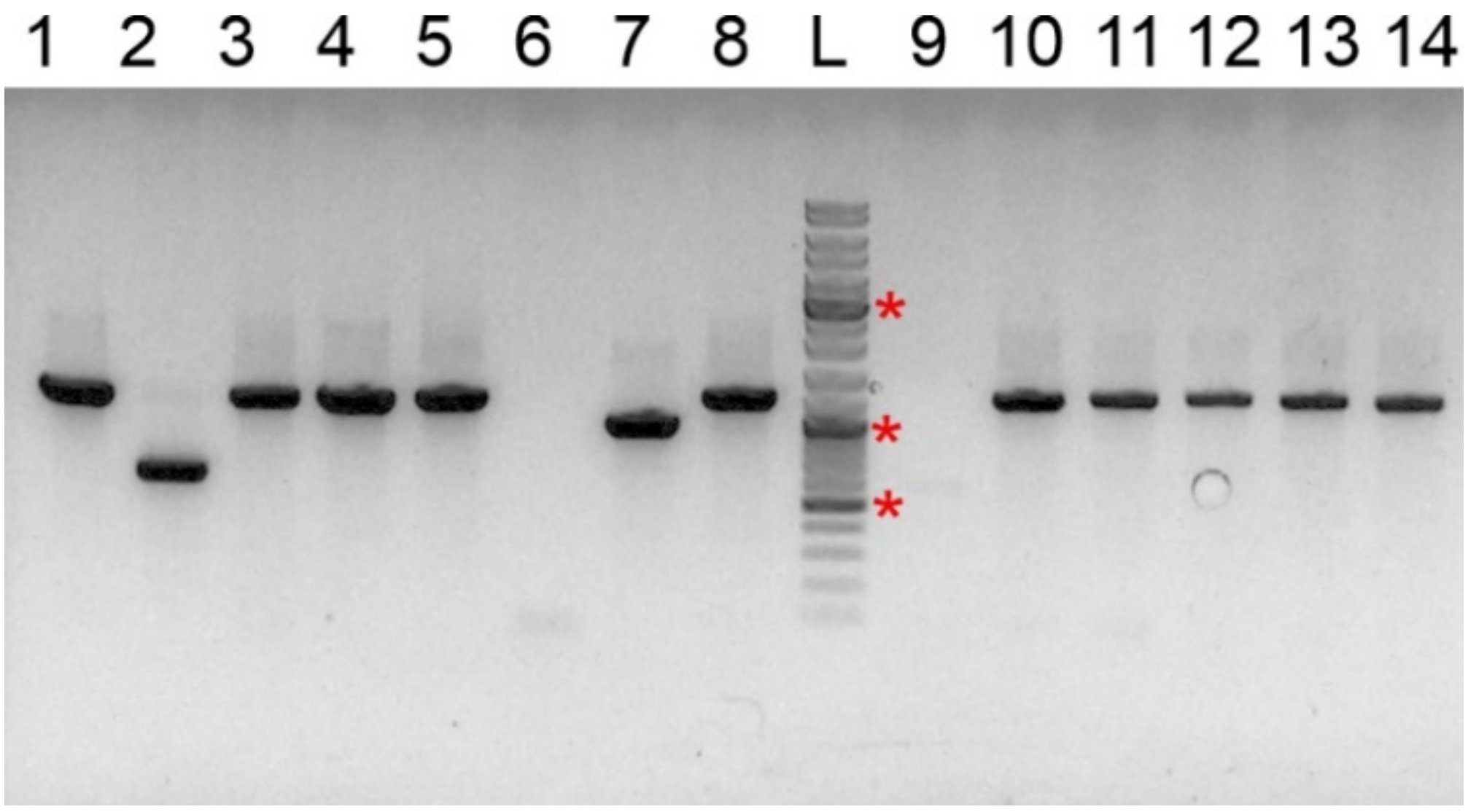

Fig. S1: PCR analysis of plaques obtained after CRISPY-BRED. Plaque regions were cored from soft agar overlays using a wide-bore pipette tip and resuspended in 100 μL of phage dilution buffer. 10 μL of this suspension was boiled for 10 min and used as PCR template. Primers flanking the deleted region were used (Supplementary Table S1). "L" denotes the DNA ladder. Three bands marked with "*" correspond to 0.5, 1.0, and 3.0 kb. The wild-type gene is ~1.2 kb. Lane 2 shows the isolated mutant carrying a 588-bp deletion.

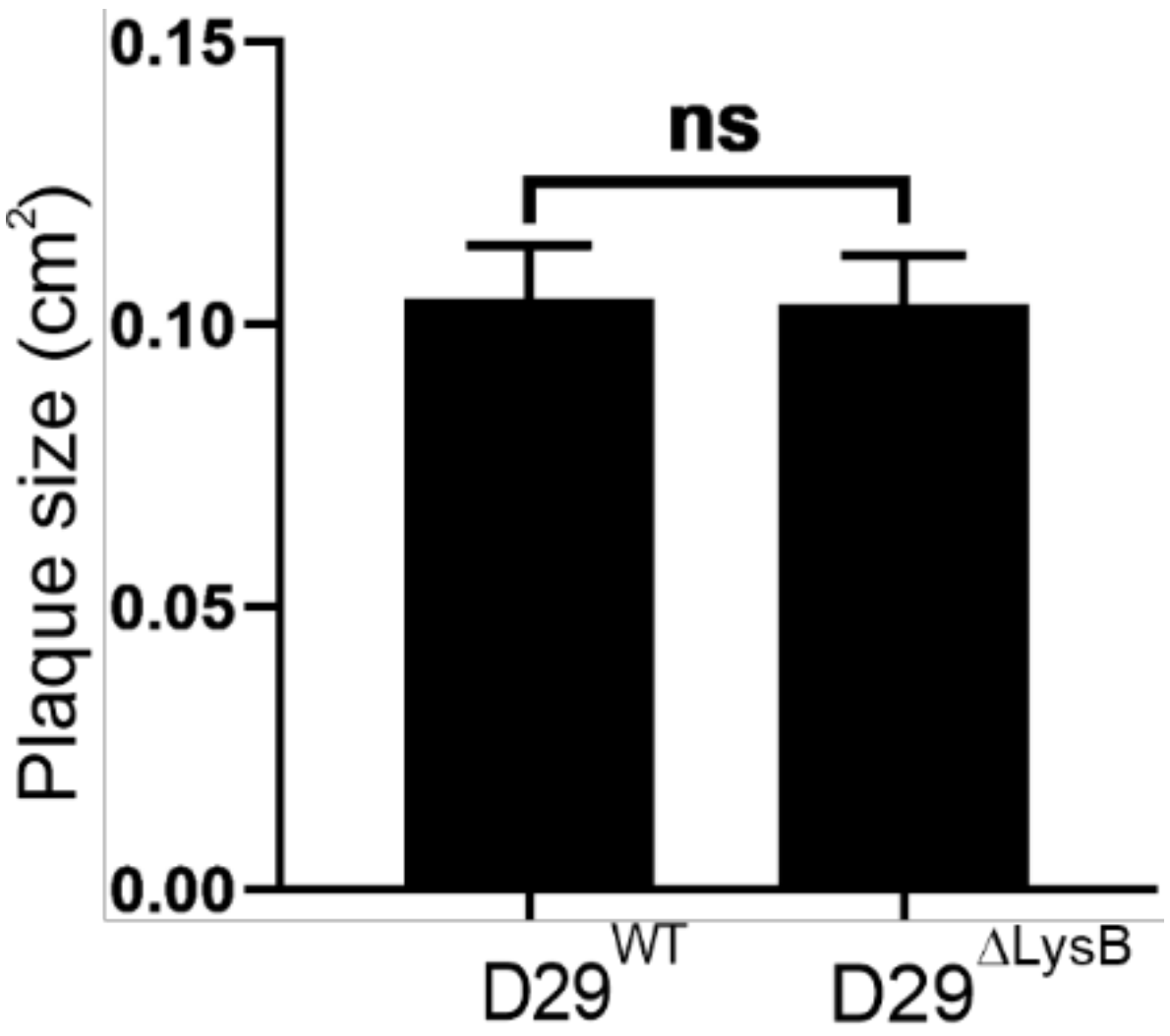

Fig. S2: Comparison of plaque sizes between D29^{WT} and D29$^{\Delta LysB}$. Average plaque areas were quantified from 50 plaques per phage, with error bars representing standard deviation. Plaque areas were measured using the *Measure* tool in ImageJ.

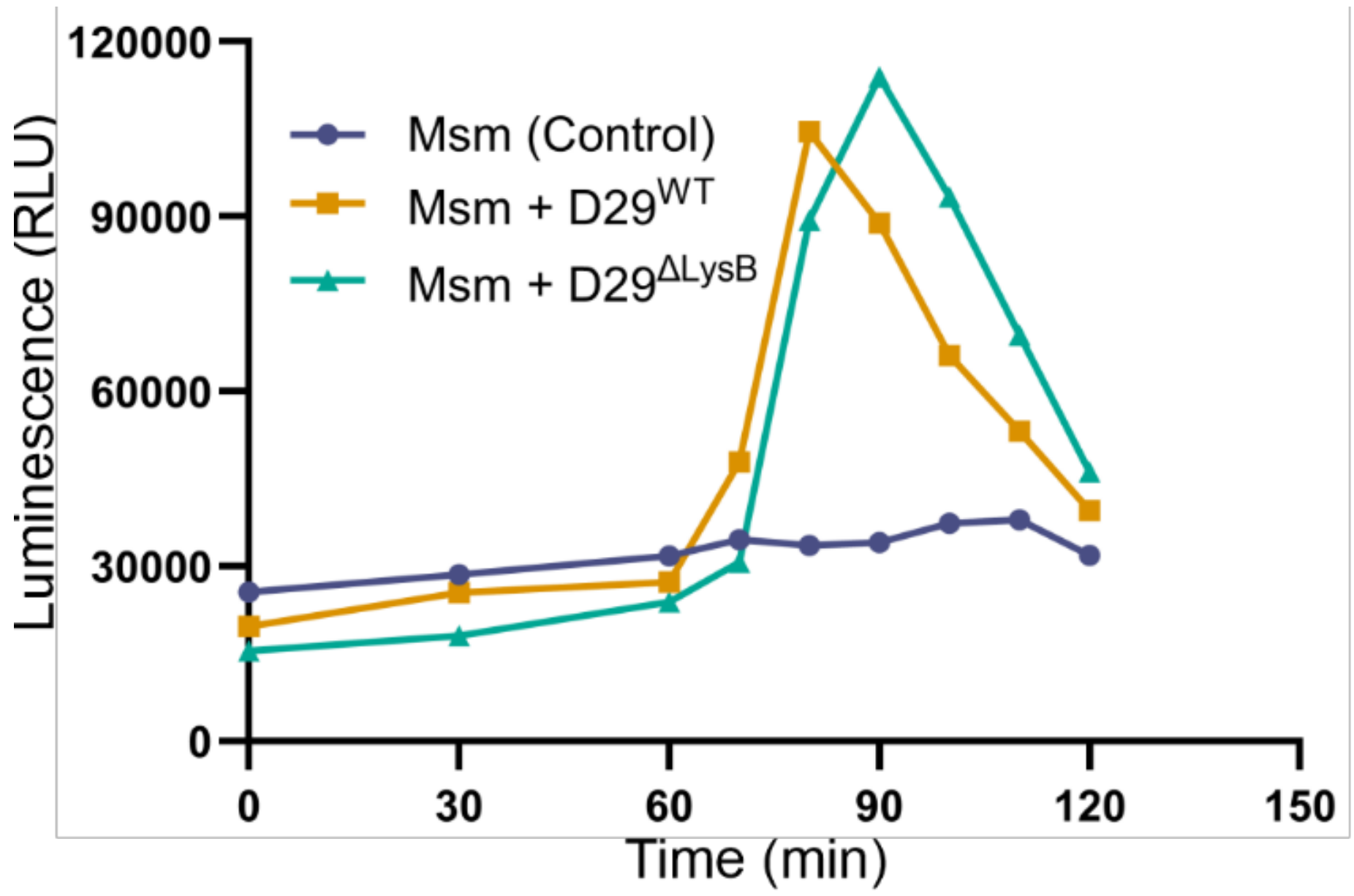

Fig. S3: ATP release following phage infection. Msm cells were infected with D29^{WT} or D29$^{\Delta LysB}$ phage at MOI = 3, and aliquots were collected at the indicated time points. ATP levels in the supernatant were measured using a luciferase-based ATP assay and are shown as relative light units (RLU). Uninfected cells served as a negative control.

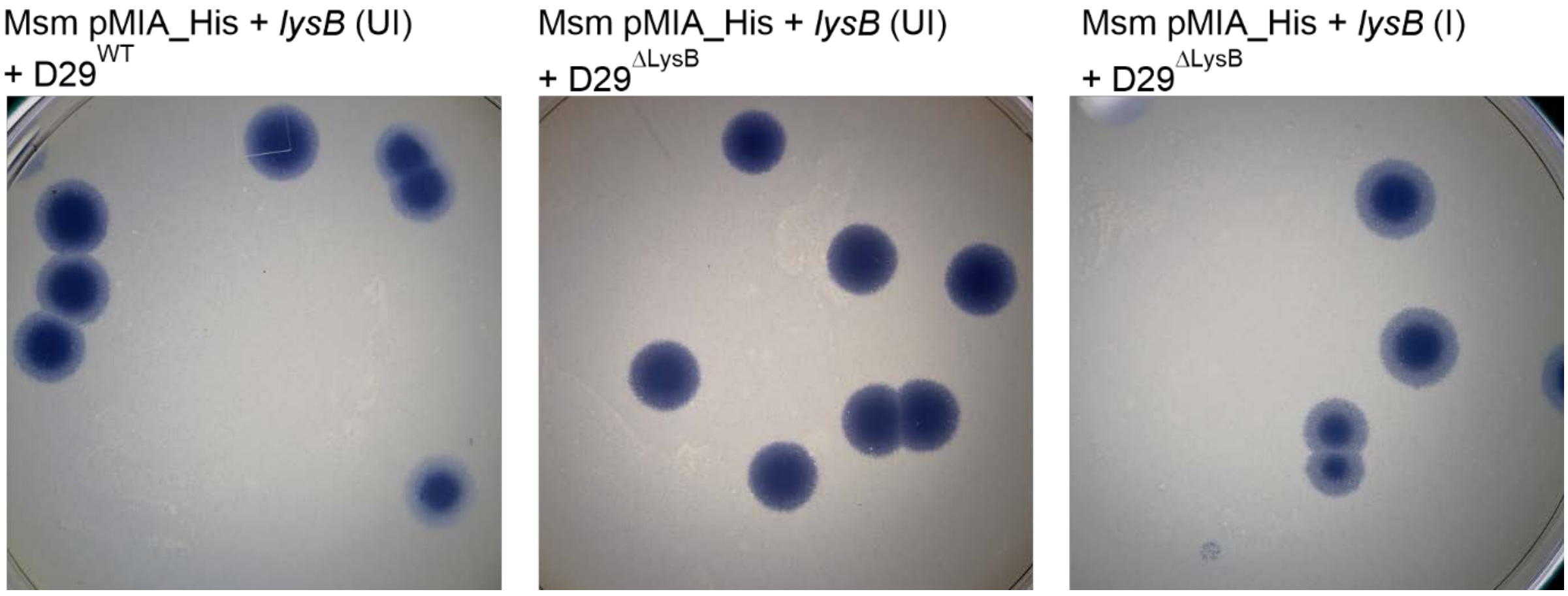

Fig. S4: Complementation of the halo phenotype in D29$^{\Delta LysB}$ plaques by host-cell expression of LysB. The *lysB* gene was cloned into the integrative vector pMIA_His under an acetamide-inducible promoter. Protein expression was induced by supplementing 0.2% acetamide in the agar. From left to right, panels show D29^{WT} propagated in uninduced Msm cells, D29$^{\Delta LysB}$ propagated in uninduced Msm cells, and D29$^{\Delta LysB}$ propagated in induced Msm cells expressing LysB. “UI” denotes uninduced, and “I” denotes acetamide-induced conditions.

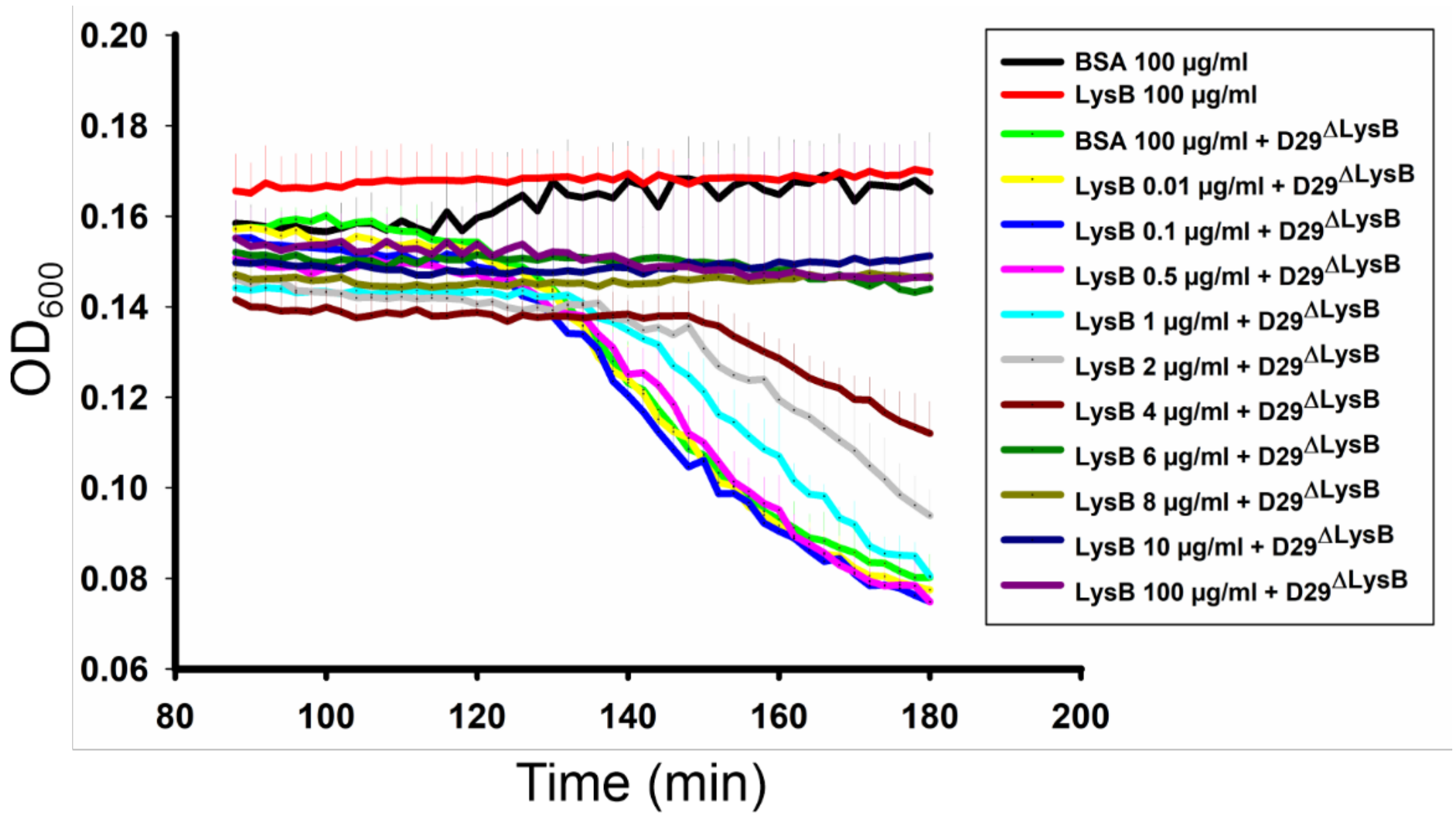

Fig. S5: Kill kinetics of LysB-treated *M. smegmatis* post phage infection. Log phase Msm cells were exposed to varying concentrations of LysB or BSA (negative control) for 30 min at 37°C. Subsequently, an infection with $D29^{\Delta LysB}$ at MOI = 3 was carried out. OD_{600} of the infected cells was measured in a multiwell plate and plotted as a function of time. Data is presented as mean ± SD of three replicates.

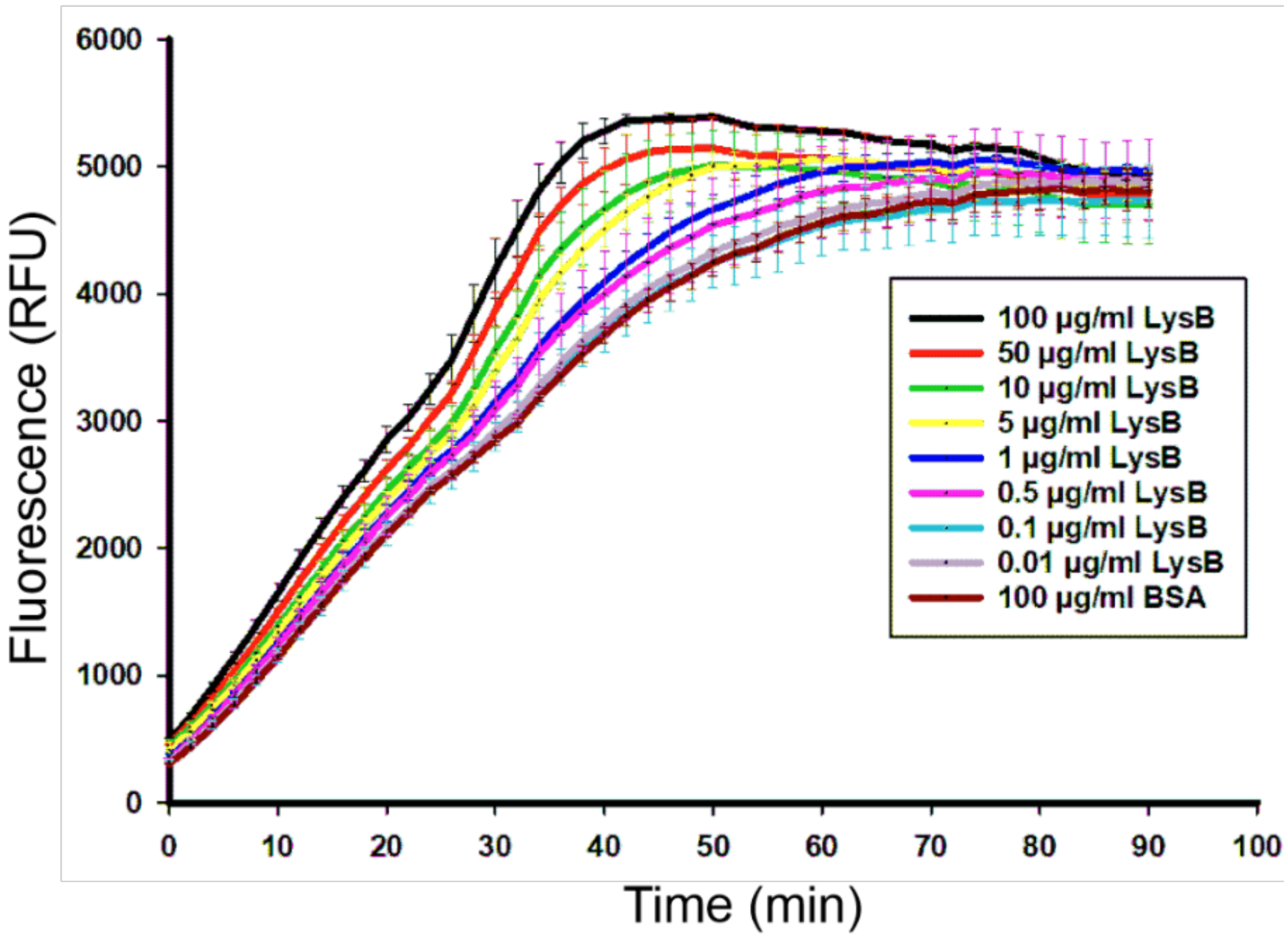

Fig. S6: Real-time alamarBlue assay post-LysB treatment. Log phase Msm cells were treated with the LysB or BSA (negative control) as indicated for 3 h at 37°C. Following this, equal amounts of alamarBlue reagent were added to all samples. Fluorescence was measured every two minutes in a multiwell plate with excitation at 530 nm and emission at 590 nm. Data is presented as mean ± SD of three replicates.

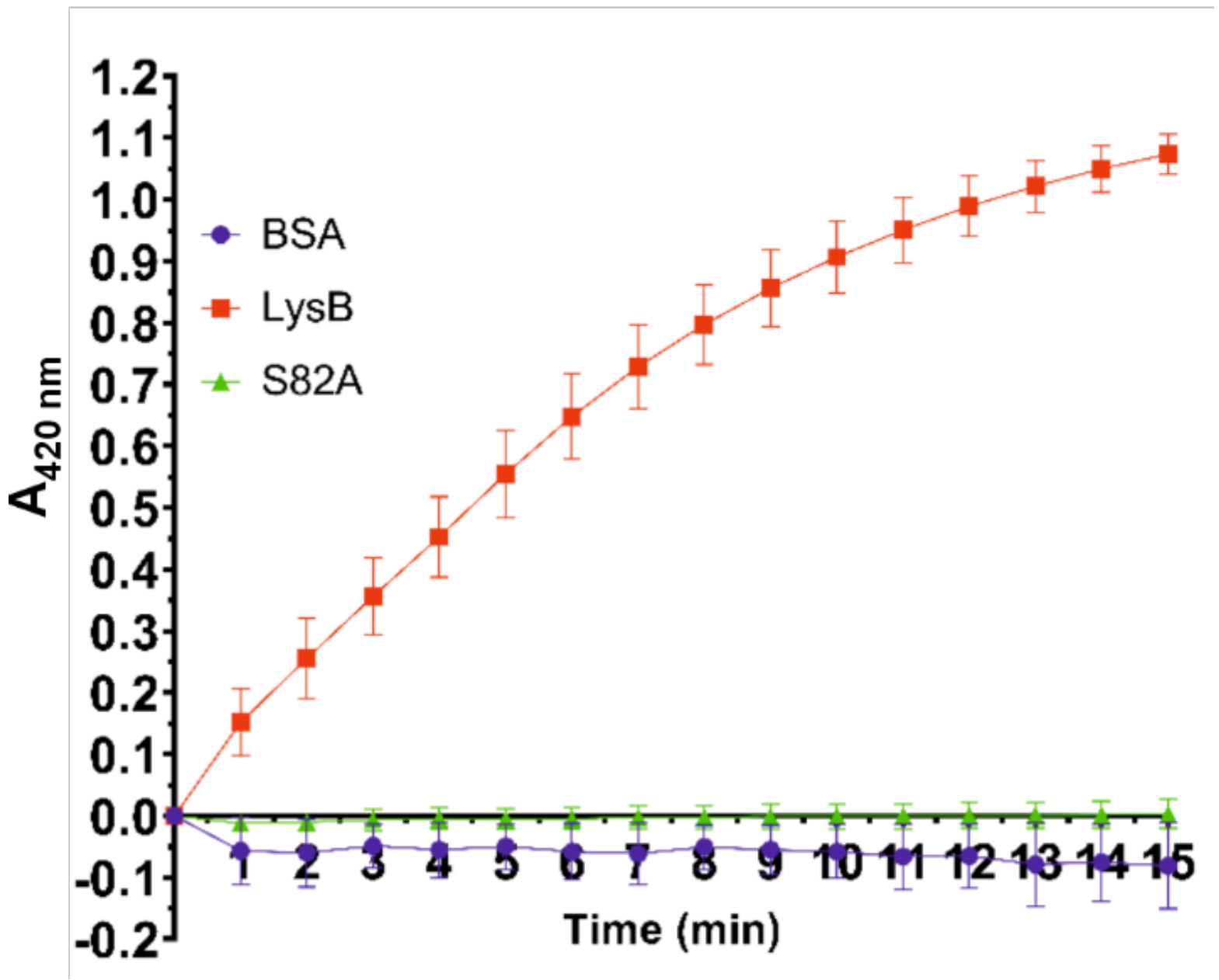

Fig. S7: Validation of the enzymatic activity of LysB and loss of activity in the S82A mutant. Catalytic activity of LysB and the S82A mutant was quantified by measuring the p-nitrophenyl release from p-nitrophenyl butyrate (p-NPB) ester substrate at 420 nm. BSA was used as a negative control. Data is presented as mean ± SD of three replicates.
