# Supplementary material for: Concentration-dependent dual activity of a phage-encoded lysin modulates infection dynamics in mycobacteriophage D29
Source: mBio. 2026 Jul 13;17(8):e00732-26. doi: 10.1128/mbio.00732-26 (PMC13463830; doi:10.1128/mbio.00732-26)
Supplement: Supplementary file 2 [file mbio.00732-26-s0002.pdf]

Supplementary tables

Table S1: List of primers used in this study. Nucleotide sequences and target reactions are indicated.

Name	Sequence (5'-3')	Purpose
D29_12For	ATGAGCAAGCCCTGGCTGTTCA	Amplification of *lysB* from D29 genome
D29_12Rev	GATCTGTCGTAGGAACTCGACC	Amplification of *lysB* from D29 genome
gp12_up_for	GGTCAACCAGCAGCGCAAGGATGGC	Generation of Recombineering substrate for CRISPY BRED - Amplification of upstream fragment, Screening of plaques after CRISPY-BRED
gp12_up_rev	GTGCGGCCACTTGTCGCTCATCGGTTCCAGGGCTGCGTGTGC	Generation of Recombineering substrate for CRISPY BRED - Amplification of upstream fragment
gp12_down_for	GCAGCCCTGGAACCGATGAGCGACAAGTGGCCGCACCTCTACAACC	Generation of Recombineering substrate for CRISPY BRED - Amplification of downstream fragment
gp12_down_rev	TGTCCCCCAGGGGTATTGACGTACTCCG	Generation of Recombineering substrate for CRISPY BRED - Amplification of downstream fragment
gp12_sgRNA_for	GGGATTCTGGGGTAATCCCATGCG	Insertion of guide RNA in pCRISPRx-Sth1Cas9-L5
gp12_sgRNA_rev	AAACCGCATGGGATTACCCCAGAA	Insertion of guide RNA in pCRISPRx-Sth1Cas9-L5
Holin_RT_For	GAGCCCCAAGATCCGTGAAACG	Screening of plaques after CRISPY-BRED

gp12_S82A	GATGGCGGGTTACGCCCAGGGAGCGATCGTGGTTGGCCAG	Primer for site directed mutagenesis (S82A) of LysB

Table S2: List of plasmids used in this study, with details of the backbone, replicon type, promoter, tag, and purpose of each construct.

Construct Name	Backbone	Replicon	Promoter	Tag	Purpose
pET-21b_lysB	pET-21 b	ColE1	T7	6xHis	Expression and purification of LysB in *E. coli*
pET-21b_lysB(S82A)	pET-21 b	ColE1	T7	6xHis	Expression and purification of LysB(S82A) in *E. coli*
pCRISPR+gRNA	pCRISPRx-Sth1Cas9-L5	Mycobacterial-Integrative	Tetracycline inducible promoter	None	Expression of Cas9 protein and guide RNA in the CRISPY BRED counterselection strain
pJV53_Hygro	pJV53	Mycobacterial-Episomal	Acetamidase promoter	None	Expression of Che9c genes 60-61 in the CRISPY BRED recombineering strain
pMIA_GFP	pMIA_GFP	Mycobacterial-Integrative	Acetamidase promoter	GFP	Inducible expression of GFP in *M. smegmatis* for GFP release assay
pMIA_His_lysB	pMIA_His	Mycobacterial-Episomal	Acetamidase promoter	6xHis	Inducible expression of LysB in *M. smegmatis* for complementation of LysB mediated halo formation
pMIT2_His_lysB	pMIT2_His	Mycobacterial-Integrative	Tetracycline inducible promoter	6xHis	Inducible expression of LysB in *M. smegmatis* for complementation of LysB mediated halo formation
